# High-*Q* dark hyperbolic phonon-polaritons in hexagonal boron nitride nanostructures

**DOI:** 10.1515/nanoph-2020-0048

**Published:** 2020

**Authors:** Georg Ramer, Mohit Tuteja, Joseph R. Matson, Marcelo Davanco, Thomas G. Folland, Andrey Kretinin, Takashi Taniguchi, Kenji Watanabe, Kostya S. Novoselov, Joshua D. Caldwell, Andrea Centrone

**Affiliations:** Physical Measurement Laboratory, National Institute of Standards and Technology, 100 Bureau Dr., Gaithersburg, MD, 20899, USA; Maryland Nanocenter, University of Maryland, College Park, MD, 20742, USA; Physical Measurement Laboratory, National Institute of Standards and Technology, 100 Bureau Dr., Gaithersburg, MD, 20899, USA; Maryland Nanocenter, University of Maryland, College Park, MD, 20742, USA; Department of Mechanical Engineering, Vanderbilt University, 101 Olin Hall, Nashville, TN, 37212, USA; Physical Measurement Laboratory, National Institute of Standards and Technology, 100 Bureau Dr., Gaithersburg, MD, 20899, USA; Department of Mechanical Engineering, Vanderbilt University, 101 Olin Hall, Nashville, TN, 37212, USA; School of Physics and Astronomy, University of Manchester, Oxford Rd, Manchester, M13 9PL, UK; National Institute for Materials Science, 1-1 Maniki, Tsukuba, Ibaraki, 305-0044, Japan.; National Institute for Materials Science, 1-1 Maniki, Tsukuba, Ibaraki, 305-0044, Japan.; School of Physics and Astronomy, University of Manchester, Oxford Rd, Manchester, M13 9PL, UK; Chongqing 2D Materials Institute, Liangjiang New Area, Chongqing, 400714, China; Department of Mechanical Engineering, Vanderbilt University, 101 Olin Hall, Nashville, TN, 37212, USA; Physical Measurement Laboratory, National Institute of Standards and Technology, 100 Bureau Dr., Gaithersburg, MD, 20899, USA

**Keywords:** dark modes, hexagonal boron nitride, high-Q, hyperbolic phonon polariton, PTIR, s-SNOM

## Abstract

The anisotropy of hexagonal boron nitride (hBN) gives rise to hyperbolic phonon-polaritons (HPhPs), notable for their volumetric frequency-dependent propagation and strong confinement. For frustum (truncated nanocone) structures, theory predicts five, high-order HPhPs, sets, but only one set was observed previously with far-field reflectance and scattering-type scanning near-field optical microscopy. In contrast, the photothermal induced resonance (PTIR) technique has recently permitted sampling of the full HPhP dispersion and observing such elusive predicted modes; however, the mechanism underlying PTIR sensitivity to these weakly-scattering modes, while critical to their understanding, has not yet been clarified. Here, by comparing conventional contact- and newly developed tapping-mode PTIR, we show that the PTIR sensitivity to those weakly-scattering, high-Q (up to ≈280) modes is, contrary to a previous hypothesis, unrelated to the probe operation (contact or tapping) and is instead linked to PTIR ability to detect tip-launched dark, volumetrically-confined polaritons, rather than nanostructure-launched HPhPs modes observed by other techniques. Furthermore, we show that in contrast with plasmons and surface phonon-polaritons, whose *Q*-factors and optical cross-sections are typically degraded by the proximity of other nanostructures, the high-*Q* HPhP resonances are preserved even in high-density hBN frustum arrays, which is useful in sensing and quantum emission applications.

## 1 Introduction

The coupled excitation of light and coherent charge oscillations in materials, known as polaritons, enables squeezing light to deeply sub-diffractional dimensions. This is paramount in the mid-IR, where device sizes are otherwise limited by diffraction to several μm, thus precluding many nanophotonic applications [1]. For most materials, polaritons are surface-bound. However, polaritons in highly anisotropic materials, where the permittivity along one of the axis is of opposite sign to the others, instead propagate within the volume of the material at an angle dictated by the ratio of the permittivity values along two orthogonal directions [2-5]. Such materials are referred to as hyperbolic. This frequency-dependent, volume propagation is highly promising for applications such as on-chip photonics [6, 7] and hyperlensing [4, 5, 8-10]. However, initial attempts to harness hyperbolic properties with metal-dielectric metamaterials suffered from high losses due to the fast carrier scattering within the metallic constituents. The discovery of natural hyperbolic materials that can support hyperbolic phonon polaritons (HPhPs) [11-13] such as the 2D polar crystal hexagonal boron nitride (hBN) has provided scientists with a new paradigm to investigate the fundamental aspects and functionalities of hyperbolic media thanks to the significantly lower losses linked to the lower scattering rates of optic phonons [14].

Unlike surface polaritons, 3D-confined HPhPs offer a progression of aspect-ratio (*A*_*r*_) dependent resonances with increasing momentum (*q*) [15-17]. For example, theory predicts that spheroidal nanoparticles will confine HPhPs to deeply subdiffractional wavelengths *λ*_*p*_ = 2*π*/*q*_*m,l,n*_, with momenta characterized by three discrete quantum numbers (*l*, *m*, *n*). In such notation, *l* is the orbital angular momentum, *m* is the azimuthal angular momentum, and *n* the radial index [17], see Figure S1 for a schematic illustration. Notably, modes with *n* > 0 display a strong field confinement within the particle volume, while for modes with *n* = 0 the field is strongest in proximity of the particle surface (see Figure S1). Parameterization of such analytical predictions with respect to the *A*_*r*_ was found to describe HPhPs confined in hBN frustum nanostructures [4, 16] reasonably well, where:

(1) $$A_{r}=\frac{d}{h}$$

and *d* is the frustum diameter at half-height and *h* is the frustum height.

HPhPs in hBN frustum nanostructures were previously imaged using two near-field IR spectroscopic techniques based on an atomic force microscope (AFM) platform: scattering-type scanning near-field optical microscopy (s-SNOM) [15] and later via photothermal induced resonance (PTIR) [16]. While only a subset of the theoretically predicted HPhP modes[17] with quantum numbers (*l*, 1, 0) were observed in s-SNOM and nano-Fourier Transform IR (FTIR) experiments [15, 18], a plethora of additional (*l*, *m*, *n*) resonances (several with *n* > 0, particularly at higher frequencies than the 1,1,0 mode) were subsequently observed with PTIR [16]. Figure S2 provides a summary of the frequencies and *Q*-factors of polaritonic modes observed previously with those two techniques in hBN frusta. The origin for this different sensitivity is currently a topic of debate. Although both techniques [19] achieve a spatial resolution well below the diffraction limit by leveraging a sharp metallized AFM tip to aid the excitation of high-momenta optical modes and for imaging them in real space [5, 15, 16, 20-22], a clear methodological difference stands in the signal detection scheme [19]. In s-SNOM, the AFM cantilever oscillates periodically above the sample (tapping-mode) and the tip-scattered light is measured optically in the far-field. In contrast, PTIR typically operates with the AFM cantilever in contact with the sample (i. e., contact-mode) and directly mechanically transduces the photothermal expansion of the sample to measure light absorption [19]. Therefore, it was suggested [16] that the different PTIR and s-SNOM sensitivities for detecting these additional higher-order dark and weakly scattering modes may originate from the different probe operation schemes (contact vs. tapping) or from the intrinsically different detection mechanisms, which for PTIR does not require scattering towards a (s-SNOM) far-field detector. In support of the latter argument, PTIR has, for example, enabled efficient detection of dark, i. e., weakly-scattering, plasmonic modes as well [21, 23]. In nanostructures arrays, the strong (weak) scattering characteristics of bright (dark) modes typically results in strong (weak) sensitivity to the nanostructure density. Consequently, the array density can provide in principle a possible mean for distinguishing the intrinsic nature of the mode.

Here, we study both sparse and dense hBN frusta arrays and leverage the conventional contact- [24] and the newly-introduced tapping-mode [25, 26] PTIR measurement modalities to test the prior hypothesis and pinpoint the origin of the peculiar sensitivity to elusive HPhP modes via PTIR. The demonstration of nominally identical results, regardless of the probe operation (tapping vs. contact modes), rules out the AFM probe operation as the reason for the discrepancies between s-SNOM [15] and PTIR measurements [16]. Theoretical modeling of the PTIR near-field response as a function of the distance between the tip and the frustum surface also shows distance-independent HPhP excitation, well-correlated with our experimental observations. Furthermore, PTIR measurements show that high-*Q* (up to ≈280) HPhP resonances are preserved, even in high density hBN frusta arrays, especially for the weakly-scattering modes observed at high frequencies (*n* > 0). Such observations contrast with the typical behavior of plasmonic and surface phonon polariton (SPhP) resonances, where the *Q*-factor and the optical cross-section are typically degraded by the proximity of other nanostructures [27, 28]. We attribute such key differences to the progressively stronger confinement of the higher momenta HPhPs, particularly the ones with *n* > 0, within the volume of the frusta, which reduces the influence of environmental permittivity and inter-particle coupling on the *Q* [29]. In general, optical excitations in nanostructures decay either by scattering in the far-field or decay into heat, [68] with relative contributions depending on the specific resonance. Since dark and weakly-scattering resonances typically have higher *Q*s than bright modes resulting from the reduced radiative contribution to the scattering lifetime, our measurements suggest that PTIR direct near-field transduction of the volumetric light-induced photothermal expansion is the likely enabling reason that permits a more complete characterization of polaritons than with s-SNOM (far-field detection of scattered light). We believe that the combination of high-*Q* and of high densities of nanostructures demonstrated in this work, holds great promise for applications in sensing and quantum emission, while also demonstrating the emerging potential of PTIR as a novel tool for characterizing nanophotonic media in the IR.

## 2 Materials and methods

### 2.1 Sample fabrication

hBN crystals were grown using a high-pressure/high-temperature method [30, 31], with hBN flakes exfoliated and transferred either onto quartz (sample-1) or intrinsic silicon (sample-2 and 3) substrates, coated with a bilayer of PMMA and patterned with electron beam lithography using an Al hard mask created via electron-beam evaporation and liftoff. Finally, the hBN nanostructures were fabricated via reactive ion etching in an oxygen environment. The residual metal was then removed with wet chemical etchants. Further details of the fabrication process are available elsewhere [32, 33].

**Figure 1:**
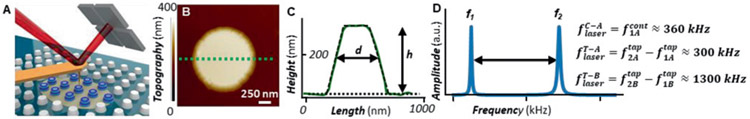
(A) PTIR schematic. A pulsed tunable IR laser illuminates the portion of the sample (region highlighted in yellow centered around gold-coated AFM probe). (B) AFM topography image of a hBN frustum with *Ar*_*1*_ = 4.65 ± 0.11 (sample-1). (C) AFM topography line profile corresponding to the green line in panel-B, showing the results of the fitting for determining the frustum dimensions. (D) Schematics highlighting the cantilever resonant frequencies and the laser repetition rate for three resonance-enhanced PTIR measurement schemes used in this work. 1. Using probe A in contact-mode the laser repetition rate ($f_{laser}^{C-A}$) matches the frequency of oscillation to the first cantilever contact-mode ($f_{1A}^{cont}\approx 360\;\text{kHz}$). 2. Using probe A in tapping-mode the probe is tapped at the first mode ($f_{1A}^{tap}\approx 55\;\text{kHz}$), the PTIR signal is demodulated at the second mode ($f_{2A}^{tap}\approx 355\;\text{kHz}$) and the laser repetition rate ($f_{laser}^{T-A}$) matches the frequency difference between the two modes (≈300 kHz). 3. With probe B in tapping-mode the probe is tapped at the second tapping mode ($f_{2B}^{tap}\approx 1550\;\text{kHz}$), the PTIR signal is demodulated at the first mode ($f_{1B}^{tap}\approx 255\;\text{kHz}$) and the laser repetition ($f_{laser}^{T-B}$) rate matches the frequency difference between the two modes (≈1300 kHz). This kind of heterodyne detection used for the tapping mode experiments is made possible by the non-linear tip-sample interactions [67] that enable mixing of the cantilever motion with the sample photothermal expansion [25, 35, 36].

**Figure 2:**
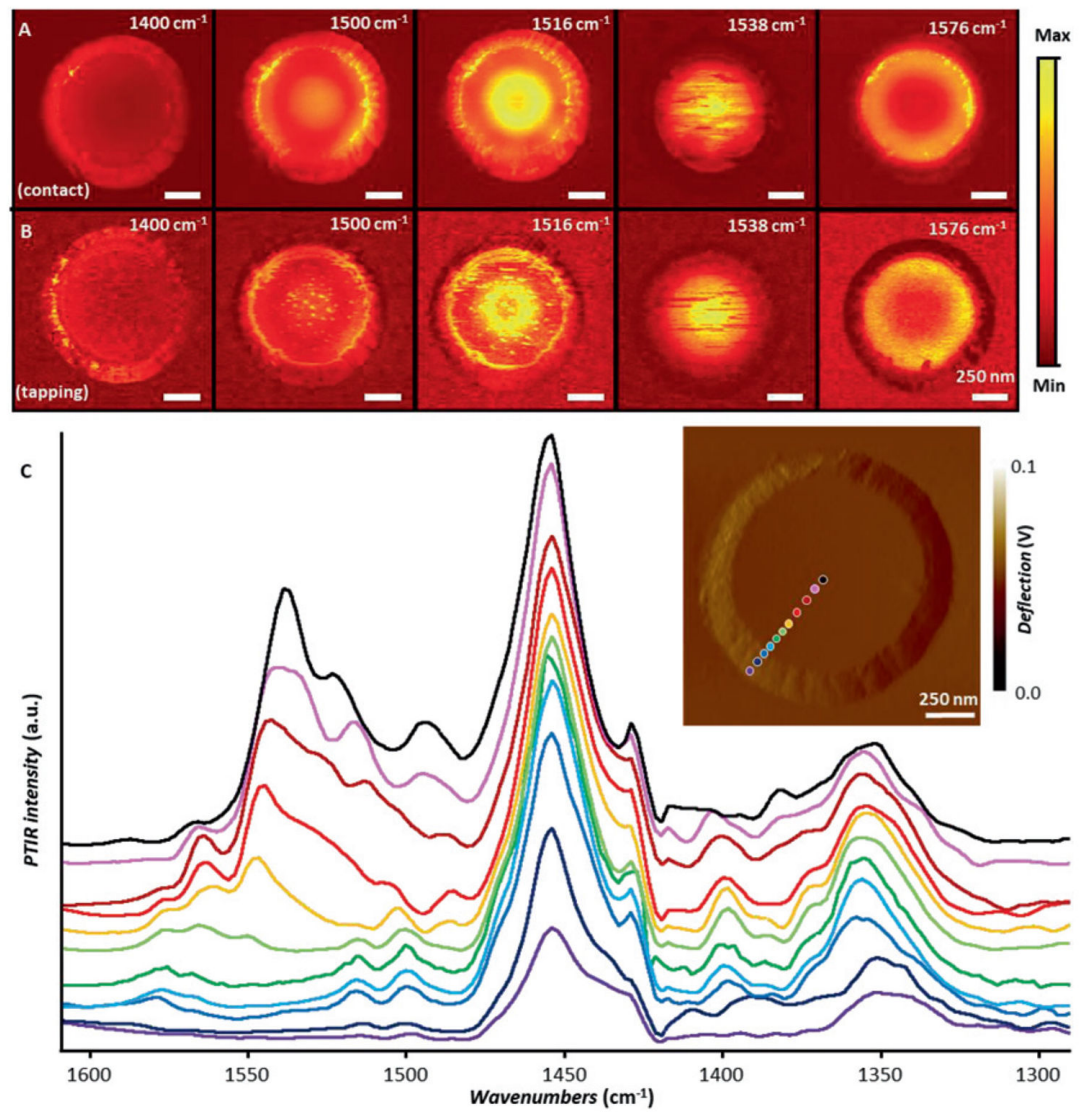
(A) Contact-mode and (B) tapping-mode PTIR absorption maps for an hBN frustum of *A*_*r*_ = 4.65 ± 0.11. All images (5 nm pixel resolution) were obtained with a scan rate of 0.5 Hz using the same AFM cantilever (probe-A). (C) Contact mode PTIR absorption spectra obtained at the color-coded positions marked in the inset. The spectra were smoothed by considering seven adjacent points and are displayed in common scale and with an offset for clarity. The inset displays the AFM deflection image of the frustum. Scale bars are 250 nm.

**Figure 3:**
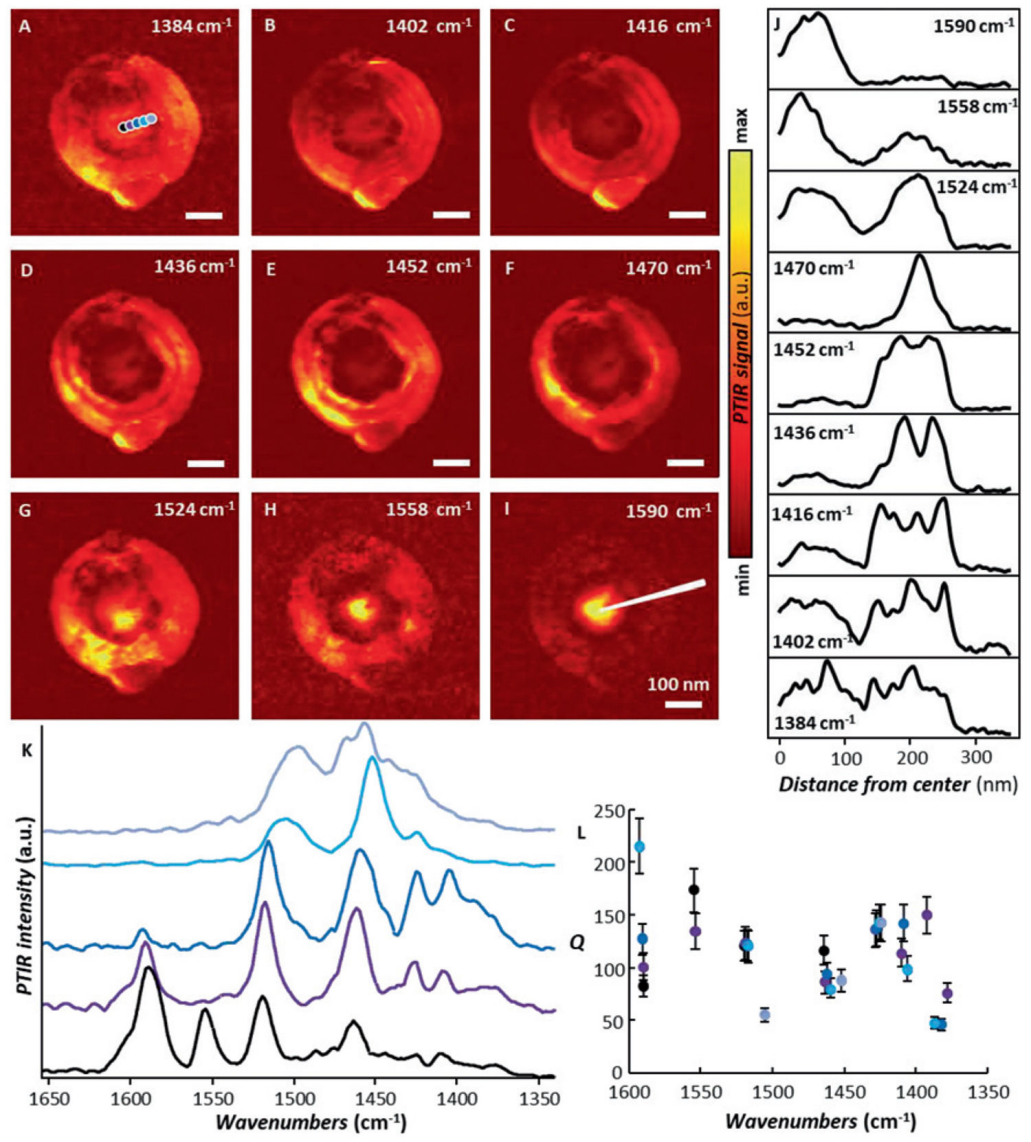
(A–I) Tapping-mode PTIR absorption maps for an hBN frustum of Ar = 1.16 ± 0.03. All images (3 and 7.5 nm pixel resolution in the horizontal and vertical directions respectively) were obtained with a scan rate of 0.6 Hz using probe-B. Scale bars are 100 nm. (J) Line cuts of the PTIR images along the direction indicated by the white line in panel-I. (K) Tapping-mode PTIR spectra obtained at the color-coded position in panel-A. The spectra are displayed in full scale with an offset for clarity. (L) Q-factors of the polariton resonances obtained from the color-coded spectra in panel-K. The error bars represent a single standard deviation in the determination of *Q* and mainly determined by the uncertainty of determining the peaks FWHM via the least square fitting the resonances with Lorentzian peak shapes.

Three samples are analyzed here. Sample-1 is characterized by *h*_*1*_ = 262 ± 1 nm and *d*_*1*_ = 1219 ± 27 nm, i. e., *Ar*_*1*_ = 4.65 ± 0.11 (see Figure 1B and C, Figure 2), while sample-2 features *h*_*2*_ = 317 ± 6 nm, *d*_*2*_ = 367 ± 8 nm, and thus an *Ar*_*2*_ = 1.16 ± 0.03 (Figure 3), with the latter representing the same hBN frusta array measured previously by s-SNOM [15]. For reference and ease of comparison Figure S3 of the supporting information displays side by side the previous s-SNOM and the new tapping PTIR maps on sample-2 obtained in this work. Sample-3 is nominally identical to sample-2, but consists of a series of adjacent frusta touching at the base (Figure 5). The uncertainties in the nanostructure dimensions represent one standard deviation in the AFM topography measurements of the same nanostructure along different directions. The nanostructure dimensions were obtained from separate linear fits of the baseline, plateau, and side walls of tapping-mode topography line cuts (Figure 1C), which provide a more accurate *A*_*r*_ estimate with respect to our previous work [16].

### 2.2 PTIR measurements

PTIR spectra and images were obtained with a commercial PTIR instrument interfaced with a wavelength-tunable quantum cascade laser that illuminates the sample from the air side at ≈20° from the sample plane, covering an area of ≈40 μm diameter centered around the AFM tip, much larger than the frusta studied here (Figure 1A). All PTIR experiments were obtained using *p*-polarization. Two distinct commercially available gold-coated silicon AFM probes were used in the experiments. Probe-A has nominal stiffness of 1–7 N/m and a nominal resonance frequency in air of 75 ± 15 kHz. Probe-B has nominal stiffness of ≈40 N/m and a nominal resonance frequency in air of ≈300 kHz. The stiffer probe-B was used in tapping-mode only (PTIR images and spectra) to measure sample-2 and 3. The softer probe-A was used to measure sample-1 both in contact-mode (PTIR spectra and images) and tapping-mode (PTIR images only). The “hybrid” mechanical characteristics of probe A are a compromise, i. e., a bit too stiff for optimal contact-and too soft for optimal tapping-mode; nevertheless, it enables a direct comparison.

In contact-mode, the resonant-excitation of the cantilever [34] is achieved by matching the PTIR laser repetition rate ($f_{laser}^{C-A}$) to one of Probe-A contact-resonance frequencies ($f_{1A}^{Cont}\approx 360\;\text{kHz}$, Figure 1D). Since the cantilever contact-resonances shift in frequency as a function of the local-tip sample interactions, to maintain the enhancement while imaging, a phase-locked loop (PLL) was employed to track the AFM tip resonant frequency and to change the *f*_*laser*_ accordingly [25].

Tapping-mode PTIR experiments with probe-A [35, 36] were obtained using a piezo actuator to drive the first mechanical resonance of the cantilever ($f_{1A}^{tap}\approx 55\;\text{kHz}$), and demodulation of the PTIR signal was performed at the second mechanical resonance ($f_{2A}^{tap}\approx 355\;\text{kHz}$). The sample was irradiated with IR pulses at a repetition rate of $f_{laser}^{T-A}=f_{2A}^{tap}-f_{1A}^{tap}\approx 300\;\text{kHz}$ (see Figure 1D).

Tapping-mode experiments with probe-B were obtained by oscillating the cantilever at its second mechanical resonance ($f_{2B}^{tap}\approx 1550\;\text{kHz}$), demodulating the PTIR signal at its first mechanical resonance ($f_{1B}^{tap}\approx 250\;\text{kHz}$), while pulsing the IR laser at $f_{laser}^{T-A}=f_{2A}^{tap}-f_{1A}^{tap}\approx 1300\;\text{kHz}$, see Figure 1D.

It should be noted that this detection scheme is very similar to the one used by photo-induced force microscopy (PiFM) [37], which measures the photoinduced force resulting from the dipole-dipole interactions between the AFM tip and the sample when illuminated by a laser. Although the origin of the PiFM signal (relative contribution of thermal expansion vs. photo-induced force) is still a subject of debate [38-41], and tapping-PTIR and PiFM appear similar, in PiFM [39, 41] the probe is tapped in the non-contact regime (attractive tip-sample force) while in tapping PTIR [25, 26, 35, 36] the tip-sample force is repulsive. PiFM was also used recently to measure HPhPs in hBN nanostructures [42].

Up to four spectra were acquired and averaged for each tip location and smoothed by considering five adjacent points unless otherwise noted. *Q*-factors were obtained by least square fitting the unsmoothed spectra using a Lorentzian peak shape. Representative fit results are provided in Figures. S4 and S5 of Supplemental Information.

### 2.3 Simulation

PTIR experiments were simulated using a finite difference time domain (FDTD) solver. The AFM tip was modeled by a discrete port mode positioned within a thin silicon shell to mitigate non-radiative coupling to the sample. This tip was positioned at various positions above the simulated frustum to determine the height and radial position dependence of the PTIR signal. The frustum was in the center of a 3 μm thick substrate, with open boundary conditions in all directions. The PTIR spectra were calculated by integrating the power loss density in the frustum at each frequency, with 0.05 cm^−1^ spectral resolution.

## 3 Results and discussion

In PTIR, as in s-SNOM, the gold-coated AFM tip (Figure 1A) aids the injection of light (*p*-polarization) into the hBN frustum, thereby bridging in momentum space the light from free space with the high momenta (i. e., small wavelength) highly confined polaritons. After excitation, the polaritons decay within a few ps into incoherent lattice vibrations (heat), leading to a temperature increase (typically <1 K) [24] and to thermal expansion (<<1 nm) [43] of the frustum during the laser pulse [44] (80–140 ns long in this work, and ≈500 ns in our previous work [16]). Subsequently, the thermal expansion decays rapidly (depending on the thickness and thermal properties of the sample) [43, 45]. The dynamics of this process can be captured directly in PTIR using fast nanophotonic probes [43]; however, the expansion is too rapid for the conventional AFM probes (tens of μs response time) [44], which, instead, are shocked by the expansion and kicked into oscillation like to a struck tuning fork. The main interest in this approach stems from the proportionality of the cantilever oscillation amplitude (measured by the AFM detector) to the absorbed energy in the sample [44, 46, 47], which enables mapping of chemical composition [26, 48, 49], molecular conformation [50] and electronic bandgap [51] at the nanoscale. As reviewed recently [19, 52], PTIR applications in material science [48, 53, 54], biology [50, 55, 56], geology [57] and other fields are growing rapidly. In the context of nanophotonics, PTIR was used to quantify the IR absorption enhancement in the near-field of plasmonic nanostructures [23, 27, 58-60], to image dark plasmonic modes,[21, 23] to identify the origin of circular dichroism in plasmonic nanostructures [22], and to characterize HPhPs in hBN frusta [16] and disks [61].

Our previous measurements of the hBN frusta were obtained with the same illumination geometry using the conventional, non-resonant PTIR excitation scheme in contact-mode, i. e., with a 1 kHz laser repetition rate, which is well below the cantilever contact resonance frequencies (typically 100–350 kHz) [16]. To increase the PTIR sensitivity, here we leverage three distinct recently introduced resonance-enhanced detection schemes for operation in contact [24, 50]- or tapping mode [25, 26, 35, 36]. Descriptions and details of the various schemes are available in the materials and methods section and Figure 1D.

For this we analyze sample-1 (*Ar* = 4.65) in both contact-mode (Figure 2A) and tapping-mode (Figure 2B) using probe-A. In contrast with previous s-SNOM images [15] that showed strong intensities only on the sidewall of the frusta (see reproduction in Figure S3b), the PTIR contact-mode images show strong intensities both on the frustum sidewall and top plateau (Figure 2A), reproducing our previous measurements on similar frusta well [16], but with 8.3-fold higher throughput thanks to the resonant detection scheme [24, 49] adopted here. The PTIR images in Figure 2A show polariton near-field patterns evolving as a function of the incident frequency, as expected for a hyperbolic medium where the polariton propagation angle is dictated by the frequency-dependent dielectric function [13]. We note that probe-A hybrid mechanical characteristics are a compromise between the contact and tapping-mode operation that results in a few speckles or streak artefacts, but nevertheless permits a direct, probe-independent comparison. Since the PTIR tapping-mode (Figure 2B) and contact-mode (Figure 2A) images on sample-1 are nearly identical, we deduce that the probe operation (contact or tapping) does not alter the PTIR response and therefore cannot be invoked to explain the differences between prior PTIR [16] and s-SNOM measurements [15]. This experiment unambiguously establishes the independence of the probe detection scheme from the ability to observe dark polaritonic modes in PTIR.

HPhPs exist only in the narrow, fixed spectral window delimited by the optic phonon pairs (Reststrahlen band) of the hyperbolic material. PTIR contact-mode spectra on the same frustum (Figure 2C) reveal several polaritonic resonances within the upper Reststrahlen band of hBN (i. e. between approximately 1610 and 1360 cm^−1^) [12, 62]. Consistent with Figure 2A and B, the PTIR spectra exhibit strong intensities on the frustum plateau, including its center, and relative peak intensities that are a function of the tip position, confirming our previous results [16], but in contrast with prior nano-FTIR spectra that were essentially invariant with the tip position [15]. Interestingly, some spectral features occur at the same frequency, independent of the spatial location of the tip (i. e., peak at roughly 1455 cm^−1^), while others shift in frequency as a function of tip position (peaks occurring at frequencies above 1550 cm^−1^). Through comparison with prior far-field results [12, 15], it is determined that the former correspond to modes that couple to the far-field (i. e., that don't require the gold tip) as suggested by the uniform near-field PTIR absorption maps (Figure S6) – see discussion later. In contrast, the spectral features that shift in frequency with probe position correspond to HPhPs with centrosymmetric near-field distributions (see Figure 2). Thus, we deduce that the AFM tip is crucial for launching at least the polaritons at frequencies above ≈1550 cm^−1^ that are characterized by modes predominantly within the nanostructure volume, rather than at the surface. This differs from s-SNOM, where the detected modes (below 1550 cm^−1^ only) were assigned to polaritons launched by the resonant frustum cavity itself [15]. In principle, such dark-modes should also tip-launched in s-SNOM experiments but since they scatter weakly to the far-field, once excited, their energy is primarily dissipated as heat though the frustum volume, a mechanism that is more favorable for PTIR detection.

Next we leverage the lower lateral forces exerted by the AFM tip when operating in tapping-mode (probe-B), to measure sample-2 (*Ar* = 1.16, Figure 3), which is from the same array of frusta measured with s-SNOM previously [15]. We note that otherwise the smaller *A*_*r*_ frusta in sample-2 are easily damaged by the AFM tip, if this is operated in contact mode.

The tapping-mode PTIR absorption images at 1402, 1416, and 1436 cm^−1^ (Figure 3 B-D) show several concentric rings on the frustum sidewall, similar to previous s-SNOM measurements [15] (see direct comparison in Figure S3). However, in contrast to those efforts, the PTIR images in Figure 3B-D again exhibit significant near-field intensity on the plateau, thus providing a more complete characterization of the polaritonic modes. The intensity in the plateau region becomes the strongest feature for images obtained at frequencies greater than 1524 cm^−1^ (Figure 3G-I). For comparison, Figure 3J displays line cuts along the marked direction in the PTIR images (in Figure 3A-I). Previous s-SNOM images were interpreted as the result of polaritons [15] launched at the intersection of the sidewall with the frustum base and reflecting on the frustum sidewall. In PTIR, however, one would expect that for polaritons launched efficiently in the far-field, for example those launched by scattering from the frustum base, would result in a spatially homogeneous signal (see Figure S6) because the high hBN thermal conductivity and short HPhPs lifetime (<10 ps) would conduce to homogeneous frustum heating during the much longer (80 ns) laser pulses. Consequently, the polaritons in Figure 3 must be tip-launched. Therefore, the PTIR intensity reflects the local coupling efficiency between the wavelengths in free space and the tip-launched HPhPs, leading to a strong signal when and where the HPhP near-fields are strongest. This hypothesis is corroborated by the tapping-mode PTIR spectra (Figure 3K) that reveal a series of very sharp polaritonic resonances whose relative intensities depend strongly on the AFM tip position. The *Q*-factors of many of these resonances are high (typically between 50 and 200) with the sharpest peak reaching *Q* values of ≈215, approaching those reported from far-field spectra [12] and later via s-SNOM in isotopically enriched hBN frusta [18]. Such large values of *Q* are presumably the result of the weak radiative coupling of these dark and weakly scattering HPhPs to free-space and may underline the different PTIR and s-SNOM sensitivities.

**Figure 4:**
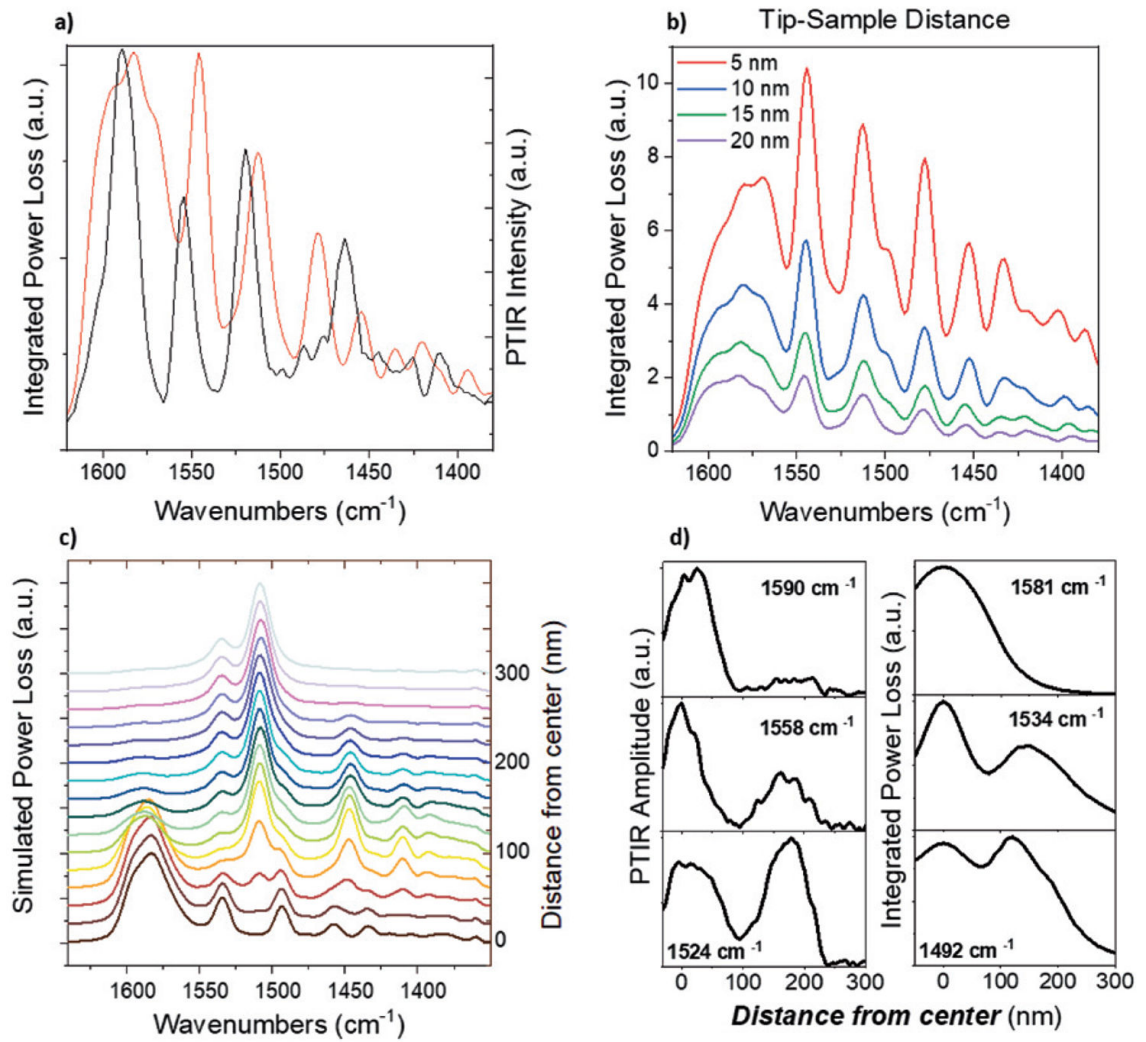
(A) Comparison of the experimental PTIR spectrum (black) and simulated power loss spectrum (red) obtained by positioning the AFM tip in the center of an isolated frustum of Ar ≈ 1.16. (B) Simulated PTIR spectra in the center of an Ar ≈ 1.16 frustum as a function of tip-sample distance. (C) FDTD integrated power loss spectra as a function of tip radial location (corresponding to Figure 3K). Spectra are shown from the center of the frustum (bottom) to 300 nm away from the center, in 20 nm increments. (D) Comparison of line cuts from PTIR scans of an Ar ≈ 1.6 frustum (Figure 3J) on the left, to the corresponding line cuts from FDTD simulations on the right, demonstrating agreement in spatial mode profile. Frequencies of line cuts are chosen for the peak center positions in the center experimental and simulated spectra identified in panel A).

We simulated the PTIR experiments on sample-2 (*Ar* = 1.16) using a finite difference time domain (FDTD) solver. By exciting the frustum with a near-field dipole source and integrating the power loss into the hBN, a similar response to the experimental observation is obtained (Figure 4A). To gauge the impact of probe operation, we varied the distance between the dipole source and the frustum surface (Figure 4B) and observed a spectral response that remains qualitatively unchanged, although with considerable change in intensity. Next, to assess the spatial distribution of the modes we radially translated the dipole source outwards from the frustum center (Figure 4C and D). Comparison with the line cuts and spectra in Figure 3J and K, shows strong qualitative agreement to the experiments. Since the simulated spectra are obtained integrating light absorption through the full structure, this result further demonstrates that the spatial variation is due to the spatially varying tip-coupling efficiency of the incoming light rather than differences in the local thermal expansion. Overall, these results support the conclusion that the mode of probe operation cannot explain the different PTIR and s-SNOM sensitivities to dark and weakly scattering HPhPs.

**Figure 5:**
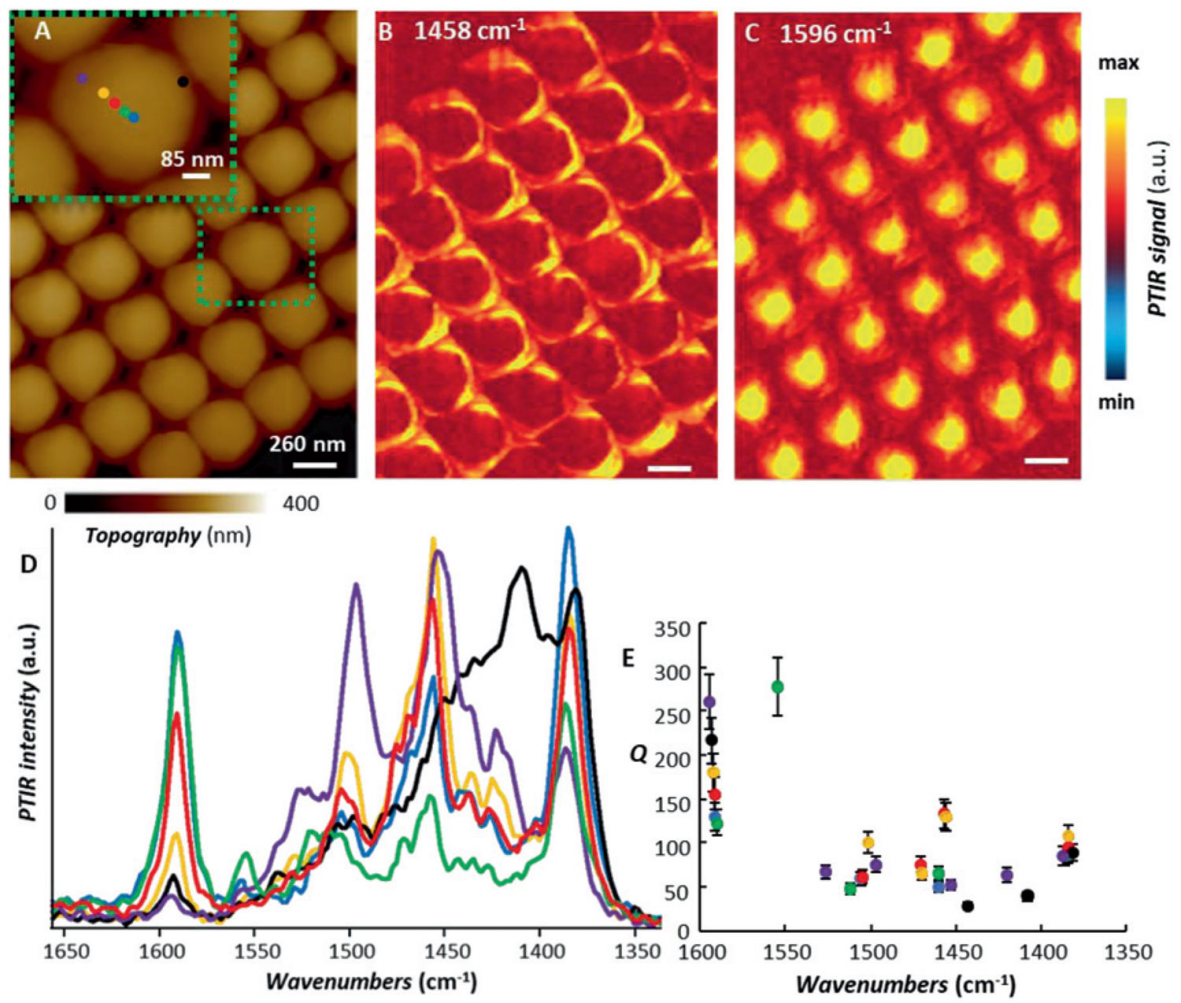
(A) sample-3 (*Ar* ≈ 1.16) AFM topography; the inset highlights the area from where the color coded PTIR spectra were obtained. (B) tapping-mode PTIR absorption map at 1458 cm^−1^ (C) tapping-mode PTIR absorption map at 1596 cm^−1^ All images (4.7 and 20 nm pixel resolution in the vertical and horizontal directions respectively) were obtained with a scan rate of 0.2 Hz using probe-B. Scale bars are 260 nm for the images and 85 nm for the inset. (D) tapping mode PTIR spectra obtained at the color-coded position marked in the inset. The spectra are displayed in common scale. (E) *Q*-factors of the polariton resonances obtained from the color-coded spectra in panel-d. The error bars represent a single standard deviation in the determination of *Q* and mainly determined by the uncertainty of determining the peaks FWHM via the least square fitting the resonances with Lorentzian peak shapes.

Next, we compare the polaritons of isolated frusta (Figure 3) with those supported in frusta fabricated in a closely packed array (Figure 5 and Figure S7). To first approximation, discounting the small geometrical irregularities, the two samples provide a qualitatively similar response. In the high-density array, for frequencies below 1550 cm^−1^ the mode intensities generally increase (particularly close to 1380 cm^−1^) while the *Q*-factors are slightly reduced. Above 1550 cm^−1^ (*n* > 0) the intensities and *Q*-factors in the high-density array are preserved if not slightly enhanced. This is in contrast with plasmonic nanostructures where the near-field is strongly influenced by the proximity of neighboring structures [27, 63]. For example, in the far-field the strongest plasmonic response is often obtained for highly packed arrays due to the higher density and in some cases due to polaritonic coupling (e. g., so-called ‘hot-spots’). However, the plasmonic response in those cases is typically accompanied by resonance broadening due to the reduction of the plasmonic cross-section, which instead is typically largest for isolated plasmonic structures [27, 64]. Our measurements however are in agreement with other observations on high-order HPhPs in hBN slabs [65], for which the field confinement inside the hBN volume progressively increases with the modal order [29]. Thus, the influence of environmental permittivity changes, i. e., due to the presence of other hBN frusta, on the resonance and field distributions is reduced [65]. This characteristic can be advantageous for enhanced spectroscopies (i. e., sensing) and thermal emitter applications as it enables significantly increased fill fractions of hyperbolic nanostructures (Figure 5) without negatively impacting the polariton *Q*-factor (see Figure S7 for a direct comparison). Preliminary observations of the PTIR near-field spectral intensity (Figure S7 and Figure 5D and E) suggests an even stronger response for the close-packed array. For example, the green spectrum in Figure 5D illustrates a polariton resonance at ≈1555 cm^−1^ with a *Q* of about 280, which is on the order of the best reported far-field values for hBN HPhPs [12], however, a proper assessment would require fabrication of the polaritonic nanostructures with more precision and less structural irregularities than those currently available.

## 4 Conclusions

We have conclusively and unambiguously identified that the mode of operation (contact or tapping) of the AFM probe is not the discriminating factor enabling the observation of dark and weakly scattering higher (*n* > 0) order HPhP modes within nanostructured hyperbolic materials. This conclusion is manifested in the PTIR spectra and maps on hBN frusta that are unchanged in character when measured in contact- or tapping-mode. Further, by employing the recently developed and gentler PTIR tapping-mode operation, we successfully measured near-field spectra and maps of the same delicate frusta originally probed by s-SNOM and nano-FTIR (also operated in tapping-mode), again clearly observing the higher order modes not detected in those samples in prior s-SNOM investigations. In general, the novel PTIR tapping mode paradigm is well suited to measure rough, delicate or sticky samples, which are notoriously difficult to measure in contact mode, with the added benefits of a bit higher (≈10 nm) spatial resolution [25, 26] than in contact-mode (≥20 nm) [24, 66]. PTIR spectra reveal polaritonic resonances with high-*Qs* (up to 280). Notably, our measurements demonstrate that the strong HPhPs volumetric confinement, particularly for the higher-*n* modes, results in a significantly decreased sensitivity to the interparticle coupling with respect to more traditional surface polaritons, a characteristic that enables preserving high-*Q* resonances even in densely packed arrays. Taken overall, our measurements suggest that the ability of the AFM probe to directly transduce the near-field absorption in PTIR experiments is likely the determining factor that enables the observation of such high-*Q* polaritonic dark resonances. We understand this as follows. Although the role of the gold-coated AFM tip in launching the polaritonic resonances in the nanostructures is the same in s-SNOM and PTIR; the detection of the near-field tip-scattered light in s-SNOM provide near-surface sensitivity to light absorption in the hBN frusta. In contrast, the thermal-expansion-based PTIR transduction provide sensitivity to light absorption within the whole nanostructure. Since for dark (*n* > 0) modes the field distribution is weak on the nanostructure surface and strong within the nanostructure volume, the PTIR technique is well suited to study these typically elusive modes. We believe that this work will stimulate research leveraging naturally hyperbolic materials for sensing and quantum emission applications, while improving our understanding of the comparative merits of these two distinct nanoprobe techniques.

## Supplementary Material

supplementary
