# Supplementary material for: High-Q dark hyperbolic phonon-polaritons in hexagonal boron nitride nanostructures
Source: Nanophotonics. Author manuscript; Available in PMC 2020 Dec 22. (PMC7754710; doi:10.1515/nanoph-2020-0048)
Supplement: supplementary [file NIHMS1642314-supplement-supplementary.docx]

**Supplementary Information for**

High-*Q* Dark hyperbolic phonon-polaritons in hexagonal boron nitride nanostructures

Georg Ramer,^1,2#^ Mohit Tuteja,^1,2#^ Joseph R. Matson,^3^ Marcelo Davanco,^1^ Thomas G. Folland,^3^ Andrey Kretinin,^4^ Takashi Taniguchi,^5^ Kenji Watanabe,^5^ Kostya S. Novoselov,^4,6^ Joshua D. Caldwell,^3^ Andrea Centrone^1*^

^1^ Physical Measurement Laboratory, National Institute of Standards and Technology, 100 Bureau Dr., Gaithersburg, MD 20899 USA
^2^ Maryland Nanocenter, University of Maryland, College Park, MD 20742

^3^ Department of Mechanical Engineering, Vanderbilt University, 101 Olin Hall, Nashville, TN 37212 USA
^4^ School of Physics and Astronomy, University of Manchester, Oxford Rd, Manchester M13 9PL, UK
^5^ National Institute for Materials Science, 1-1 Maniki, Tsukuba, Ibaraki 305-0044 Japan

^6^Chongqing 2D Materials Institute, Liangjiang New Area, Chongqing, 400714, China


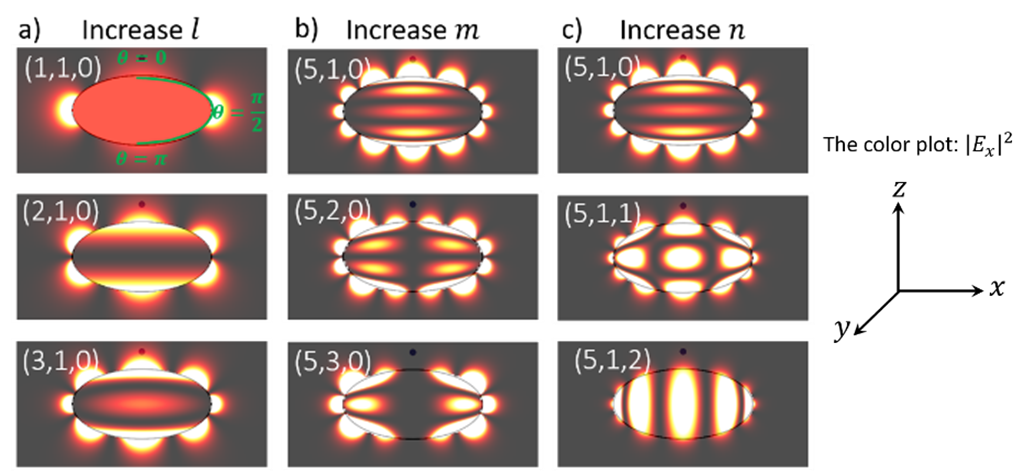
**Fig. S1:** Schematic illustrating HPhP modes of quantum numbers (*l*, *m*, *n*) in spheroidal nanoparticles. a) As *l* increases the number of nodes (=*l*-*m*) in the electric field amplitude along the θ direction (see green inset) increases. b) As *m* increases, the number of nodes in the azimuthal direction increases. c) As *n* increases, the number of nodes in the radial direction (from the spheroidal centroid to the surface) increases. Specifically, n = 0 defines as a pure surface mode, while n > 0 implies volume-confinement. Adapted with permission from (L. V. Brown, Nano Lett., 2018, 18, 1628-1636. Copyright (2018) American Chemical Society.

**
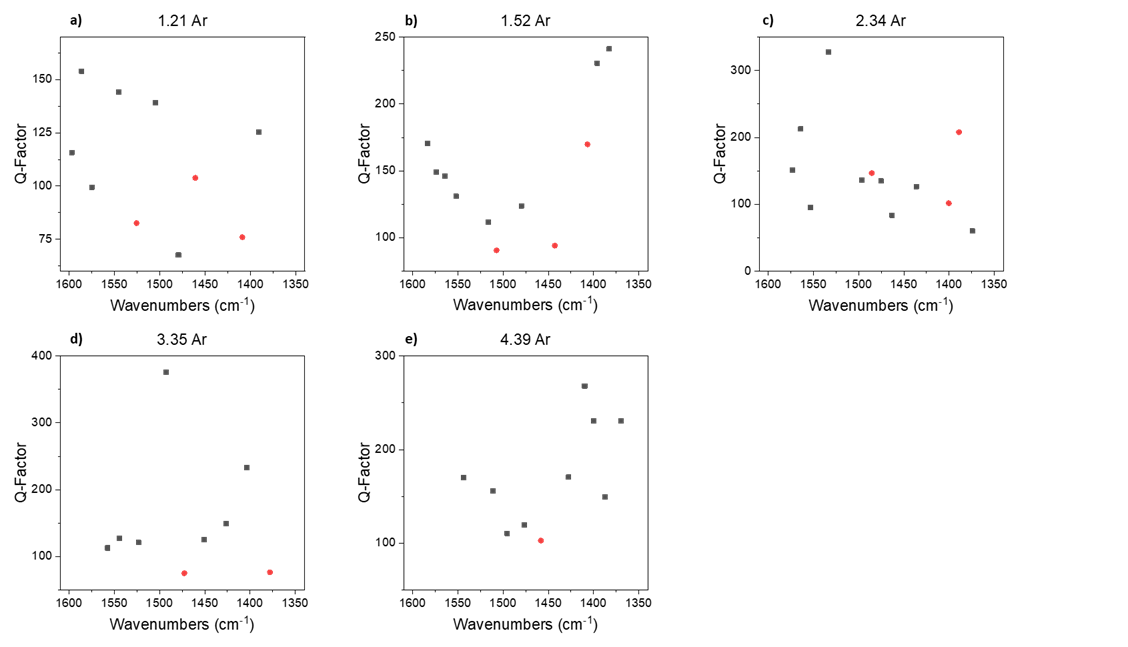
**

**Fig S2**. Comparison of the HPhP modes Q factors in hBN frusta nanostructures previously observed with PTIR (contact-mode, black), with the ones also seen in far-field and s-SNOM (red) for hBN frusta of different aspect ratios, as extracted from a previous publication.^1^


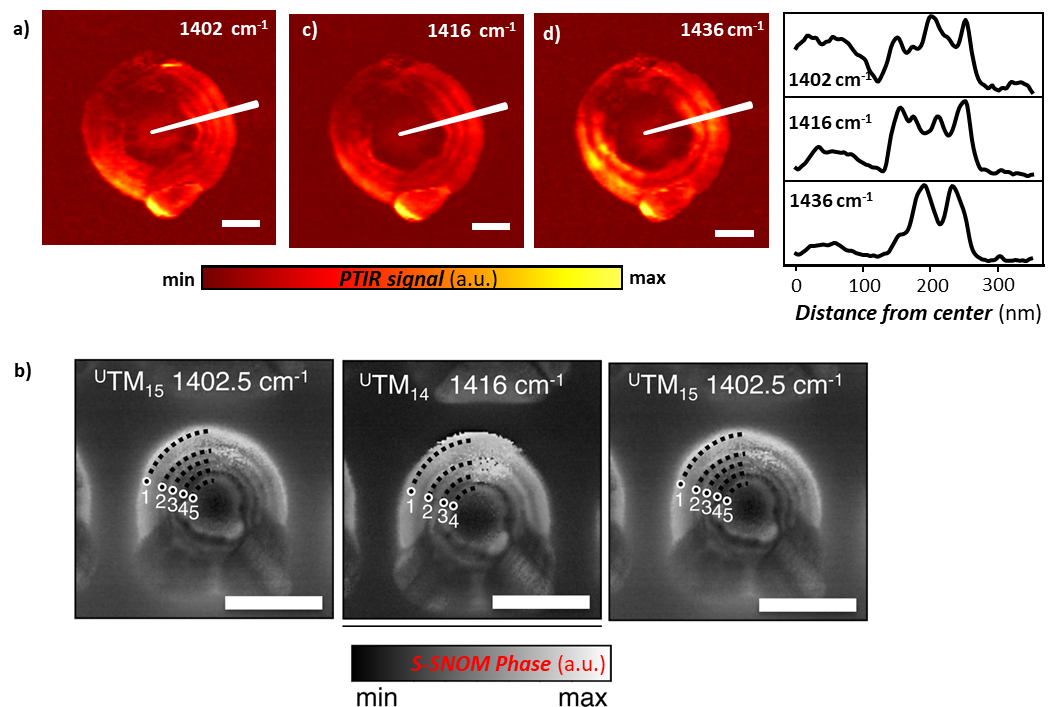


**Fig S3**. Comparison for an hBN frustum of Ar = 1.16 ± 0.03. of a) tapping-mode PTIR absorption maps (0.6 Hz using probe-B, scale bars are 100 nm) and line profiles obtained in this work (same figure 3 of the main text) and b) previously reported^2^ s-SNOM images. Adapted with permission from (A. J. Giles, Nano Lett., 2016, 16, 3858-3865. Copyright (2016) American Chemical Society.


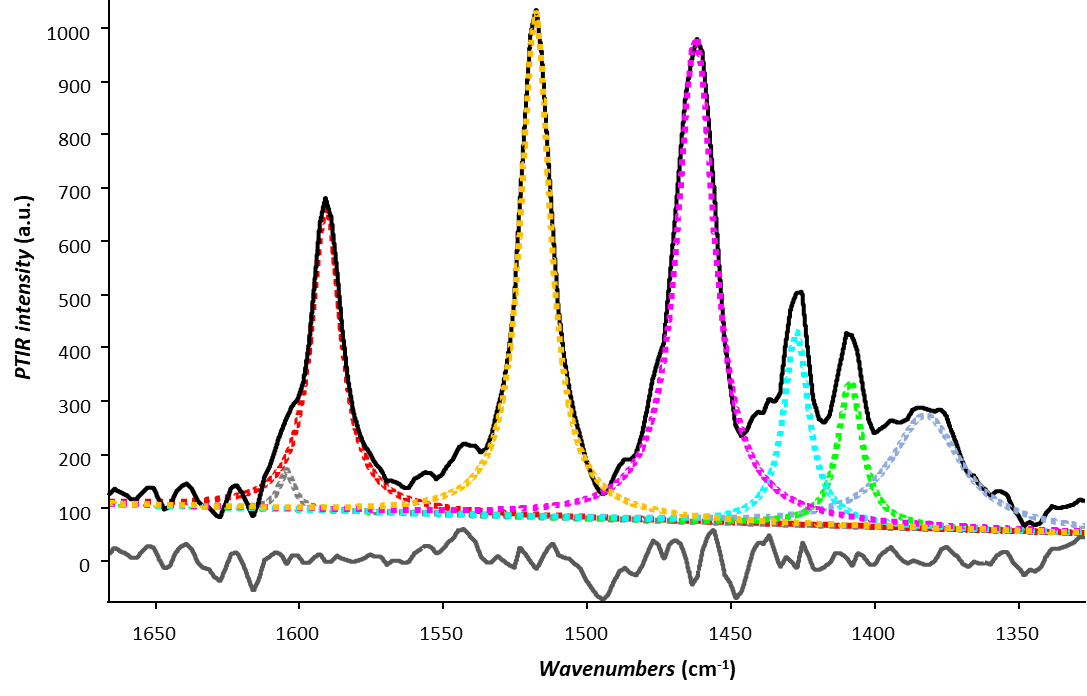


**Fig S4**. Tapping-mode PTIR spectrum (black solid line) corresponding to the middle spectrum (blue) in Fig 3k. The dotted lines show the result for the least square fitting deconvolution the resonances (Lorentzian-shape) used to calculate the resonances’ *Q* factors. The grey continuous line shows the residual of the fit displayed with an offset for clarity.


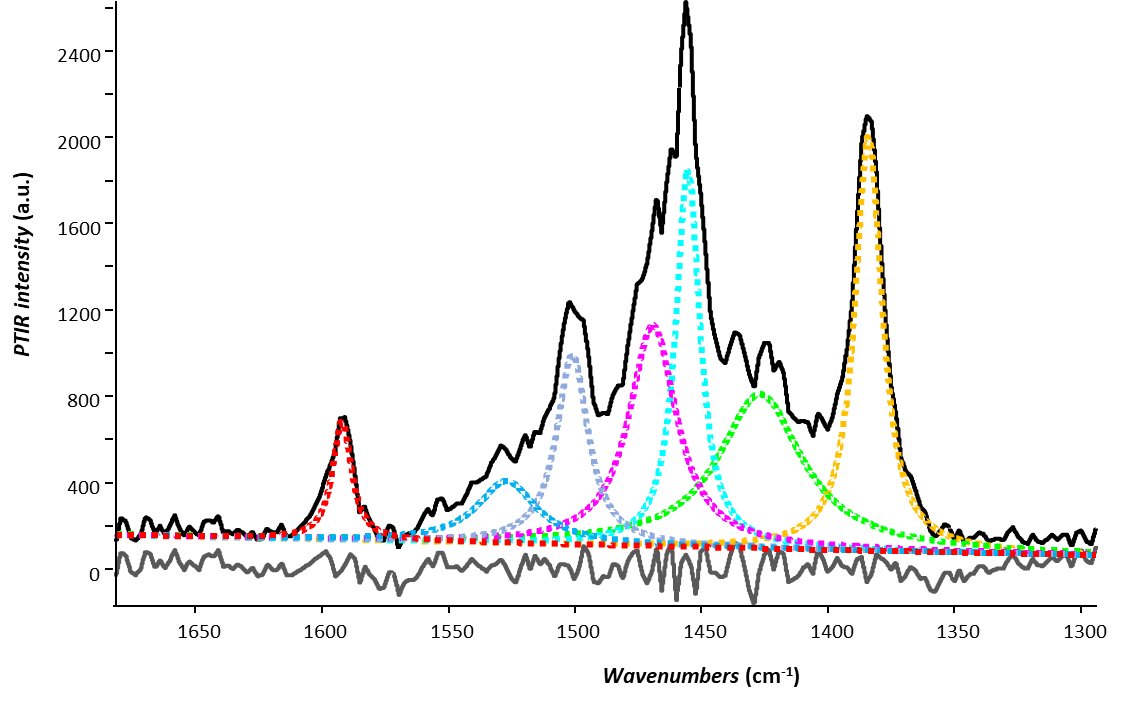


**Fig S5**. Tapping-mode PTIR spectrum (black solid line) corresponding to the yellow spectrum in Fig 5d. The dotted lines show the result for the least square fitting deconvolution the resonances (Lorentzian-shape) used to calculate the resonances’ Q factors. The grey continuous line shows the residual of the fit displayed with an offset for clarity.


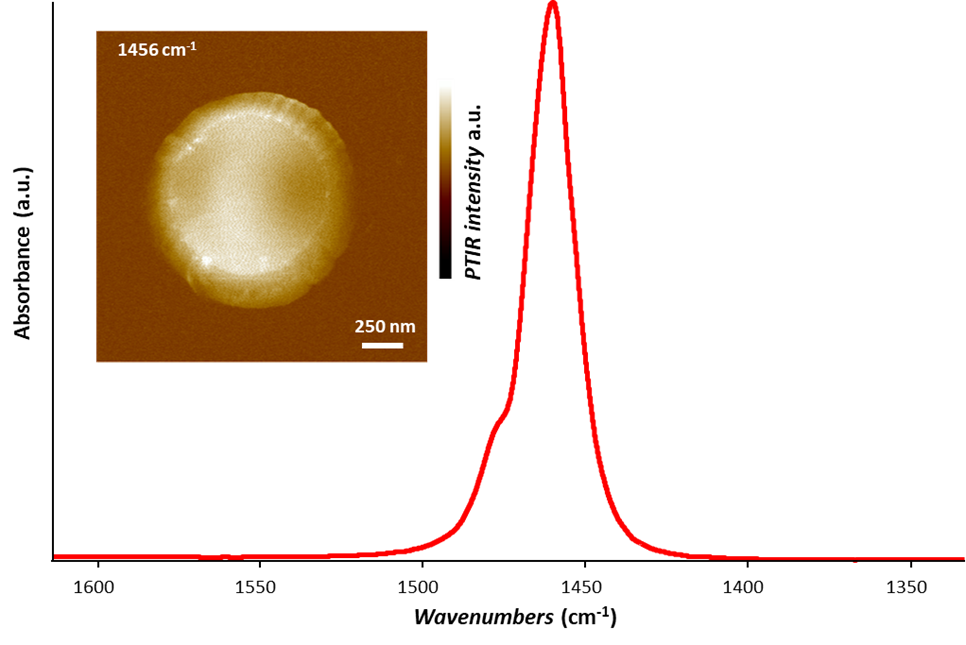
**Fig. S6:** FTIR far-field spectrum of an hBN frustum array with Ar = 4.65 ± 0.11. The inset displays the contact-mode PTIR absorption map at 1456 cm^-1^ (5 nm pixel resolution, 0.5 Hz) corresponding to the phonon polariton mode that is efficiently excited in the far field. The image was obtained using probe-A.


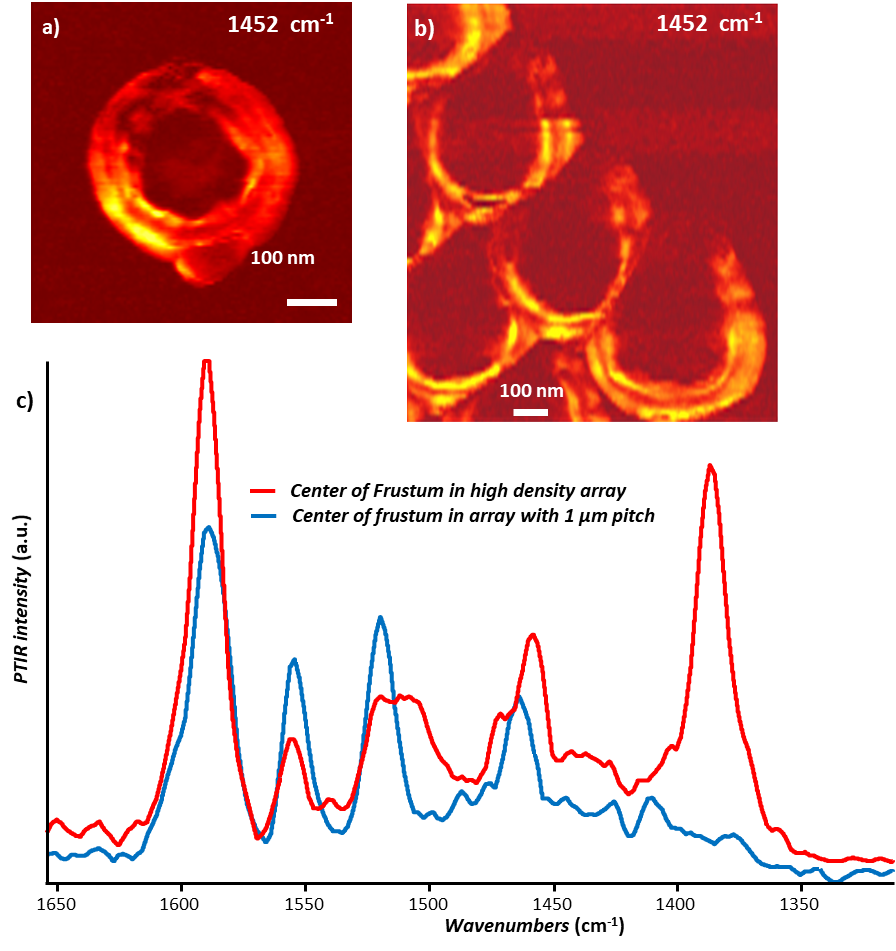


**Fig. S7**: Tapping-mode PTIR absorption maps at 1452 cm^-1^ for hBN frusta with *A_r_* ≈ 1.16 in the case of a) an isolated frustum (sample 2, array with 1 µm pitch) and b) closely packed (sample-3). The image in panel-a has a pixel resolution of 3 nm and 7.5 nm in the horizontal and vertical directions respectively. The image in panel-b has a pixel resolution of 2.75 nm and 12.6 nm in the horizontal and vertical directions respectively. Both images were obtained using probe-B. Scale bars are 100 nm. c) Tapping-mode PTIR spectra obtained in the frustum center for an isolated frustum (blue) and for a frustum in a closely packed array (red). The spectra are displayed in a common intensity scale.

**Supplementary REFERENCES**

(1) Brown, L. V.; Davanco, M.; Sun, Z.; Kretinin, A.; Chen, Y.; Matson, J. R.; Vurgaftman, I.; Sharac, N.; Giles, A. J.; Fogler, M. M.; Taniguchi, T.; Watanabe, K.; Novoselov, K. S.; Maier, S. A.; Centrone, A.; Caldwell, J. D. Nanoscale Mapping and Spectroscopy of Nonradiative Hyperbolic Modes in Hexagonal Boron Nitride Nanostructures. *Nano Lett.* **2018**, *18*, 1628-1636.

(2) Giles, A. J.; Dai, S.; Glembocki, O. J.; Kretinin, A.; Sun, Z.; Ellis, C.; Tischler, J. G.; Taniguchi, T.; Watanabe, K.; Fogler, M. M.; Novoselov, K. S.; Basov, D. N.; Caldwell, J. D. Imaging of Anomalous Internal Reflections of Hyperbolic Phonon-Polaritons in Hexagonal Boron Nitride. *Nano Lett.* **2016**, *16*, 3858-3865.
